# Topical Pimecrolimus for Treatment-Refractory Tazarotene-Induced Photosensitive Facial Erosions

**DOI:** 10.7759/cureus.106855

**Published:** 2026-04-11

**Authors:** Tara Sanjabi, Kian Memari, Sergio A Rodriguez, Joren Manuel, Peter Cohen, Shane Williams

**Affiliations:** 1 Dermatology, Touro College of Osteopathic Medicine Montana, Great Falls, USA; 2 Family Medicine, Palmetto General Hospital, Hialeah, USA; 3 Research, Nova Southeastern University Dr. Kiran C. Patel College of Osteopathic Medicine, Davie, USA; 4 Family Medicine, Nova Southeastern University Dr. Kiran C. Patel College of Osteopathic Medicine, Davie, USA

**Keywords:** calcineurin inhibitors, photodermatoses, pimecrolimus, retinoid dermatitis, tazarotene

## Abstract

Topical retinoids are cornerstone therapies in the management of acne vulgaris, psoriasis, and photoaging. Tazarotene, a third-generation retinoid, is highly effective but is associated with cutaneous adverse effects, including irritation, photosensitivity, and epidermal barrier disruption. While most retinoid-induced reactions are self-limited and respond to conservative management, a subset of patients may develop persistent inflammatory skin changes refractory to standard therapy.

We report the case of a 20-year-old man with severe acne vulgaris who developed bilateral photosensitive facial erosions following chronic tazarotene use in the setting of prolonged cumulative ultraviolet exposure. Despite discontinuation of tazarotene and extended treatment with emollients, photoprotection, and topical corticosteroids, the lesions persisted for six months. Laboratory evaluation for autoimmune and systemic inflammatory disease, including antinuclear antibody (ANA), erythrocyte sedimentation rate (ESR), and C-reactive protein (CRP), was unremarkable. Subsequent treatment with pimecrolimus 1% cream resulted in progressive clinical improvement, with complete healing of erosions within three months.

This case highlights topical calcineurin inhibition as an effective steroid-sparing strategy for persistent retinoid-associated inflammatory dermatoses and underscores the role of cumulative ultraviolet exposure as a potentiating factor.

## Introduction

Topical retinoids are foundational therapies in dermatology because they normalize follicular keratinization, reduce microcomedone formation, and exert anti-inflammatory effects, making them highly effective in the treatment of acne vulgaris and related disorders [1,2]. Tazarotene, a third-generation acetylenic retinoid, selectively binds retinoic acid receptors and is associated with potent clinical efficacy but also increased rates of local irritation, including erythema, scaling, burning, and irritant dermatitis [1,2].

Photosensitivity is a recognized adverse effect of topical retinoid therapy and may be amplified when epidermal barrier integrity is compromised or when ultraviolet exposure is substantial [1,2]. Although most reactions improve with dose adjustment, discontinuation, and photoprotection, persistent inflammatory reactions can occur and present a therapeutic challenge, particularly on facial skin, where prolonged corticosteroid use is limited by adverse effects such as atrophy and telangiectasia [3].

Topical calcineurin inhibitors such as pimecrolimus provide targeted anti-inflammatory effects by inhibiting T-cell activation without inducing epidermal thinning, making them well-suited for sensitive areas [4-6]. Their steroid-sparing properties support off-label use in selected inflammatory facial dermatoses.

We present a case of treatment-refractory tazarotene-induced photosensitive facial erosions successfully managed with topical pimecrolimus, emphasizing the interaction between chronic retinoid exposure and cumulative ultraviolet exposure.

## Case presentation

A 20-year-old man with severe acne vulgaris presented with persistent bilateral facial erosions localized to the zygomatic regions. He reported daily application of a 0.1% topical tazarotene cream for approximately four years.

The patient's occupational history was notable for prolonged and repetitive ultraviolet exposure. He had worked as a pool lifeguard every summer from age 13 through age 20, typically completing six-hour outdoor shifts daily over three consecutive months each year. During this period, photoprotection was inconsistent, particularly while continuing topical retinoid therapy.

The eruption began following prolonged sun exposure while using tazarotene, after which he developed pruritic, raised, violaceous erosions over both zygomatic prominences.

Initial management included discontinuation of tazarotene, use of bland emollients, and strict photoprotection with a broad-spectrum sunscreen and sun-avoidance counseling. Despite adherence for approximately three months, the lesions persisted.

He was subsequently treated with topical mometasone furoate 0.1% cream. Given the chronicity and atypical appearance, laboratory evaluation was performed to exclude systemic inflammatory disease. Antinuclear antibody (ANA) testing was negative, and both erythrocyte sedimentation rate (ESR) and C-reactive protein (CRP) were within normal limits (Table 1). 

**Table 1:** Autoimmune and inflammatory markers

Laboratory test	Result
Antinuclear antibody (ANA)	Negative
Erythrocyte sedimentation rate (ESR)	Within normal limits
C-reactive protein (CRP)	Within normal limits

After an additional three months of corticosteroid therapy without meaningful improvement, treatment was transitioned to pimecrolimus 1% cream applied twice daily.

**Figure 1:**
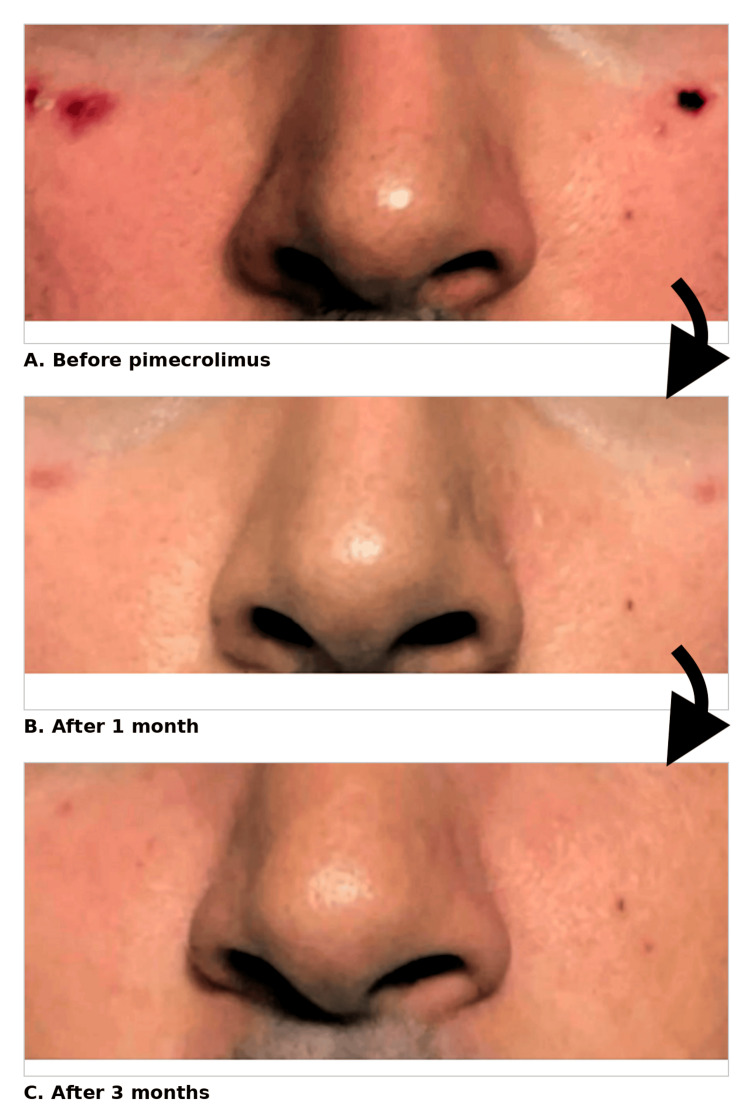
Clinical progression of bilateral zygomatic erosions before and after topical pimecrolimus (A) Baseline clinical photograph demonstrating bilateral zygomatic erosions prior to the initiation of therapy. (B) One-month follow-up showing interval re-epithelialization and reduced inflammation. (C) Three-month follow-up demonstrating complete healing with restoration of normal skin architecture. These are original clinical photographs obtained during routine patient care. Written informed consent for publication was obtained. No artificial intelligence (AI) or large language model (LLM) tools were used in the creation or modification of this figure.

Within one month, the lesions demonstrated decreased violaceous discoloration and a significant reduction in pruritus. At three months, complete re-epithelialization and resolution of inflammation were observed, with no recurrence or adverse effects (Figure 1).

## Discussion

This case illustrates a persistent photosensitive inflammatory facial eruption linked to chronic topical tazarotene use, which did not improve with standard conservative care or prolonged topical corticosteroid therapy. While topical retinoids are effective for acne vulgaris, they can cause erythema, scaling, and irritation, particularly with long-term use or exposure to ultraviolet light [1,2]. Most retinoid-related reactions are transient and respond to dose adjustments, short breaks, moisturization, and photoprotection [1,2]. However, the persistent symptoms in this patient indicate a more sustained inflammatory response than typical irritant retinization.

Prolonged sun exposure during ongoing tazarotene therapy triggered the eruption. Although patients are generally advised to avoid sun exposure and use sunscreen due to irritation and photosensitivity risks with tazarotene, significant reactions can be exacerbated by chronic barrier impairment [1,2]. The bilateral zygomatic distribution and timing of sun exposure support a diagnosis of photosensitive retinoid-associated eruption rather than an unrelated inflammatory dermatosis.

The lack of improvement with emollients, strict photoprotection, and corticosteroid therapy is clinically significant. Mometasone furoate 0.1% cream, a medium-potency corticosteroid, is less desirable for prolonged facial use due to potential adverse effects like epidermal atrophy, telangiectasia, steroid rosacea, and periorificial dermatitis [3]. The transition to a steroid-sparing anti-inflammatory strategy was necessitated not only by an inadequate therapeutic response but also by the clinical imperative to avoid cumulative corticosteroid-induced adverse effects on sensitive skin.

Topical calcineurin inhibitors, such as pimecrolimus, offer a unique approach to dermatological conditions. Pimecrolimus inhibits calcineurin-dependent activation of T lymphocytes and reduces downstream inflammatory cytokine signaling without affecting collagen synthesis or causing epidermal thinning [4-6]. This makes it ideal for long-term use on the face and thin-skinned areas, where steroid side effects can be problematic [4-6]. Notable improvements in pruritus and lesion appearance within one to three months indicate that local immune modulation significantly contributes to recovery when conventional treatments fail.

**Figure 2:**
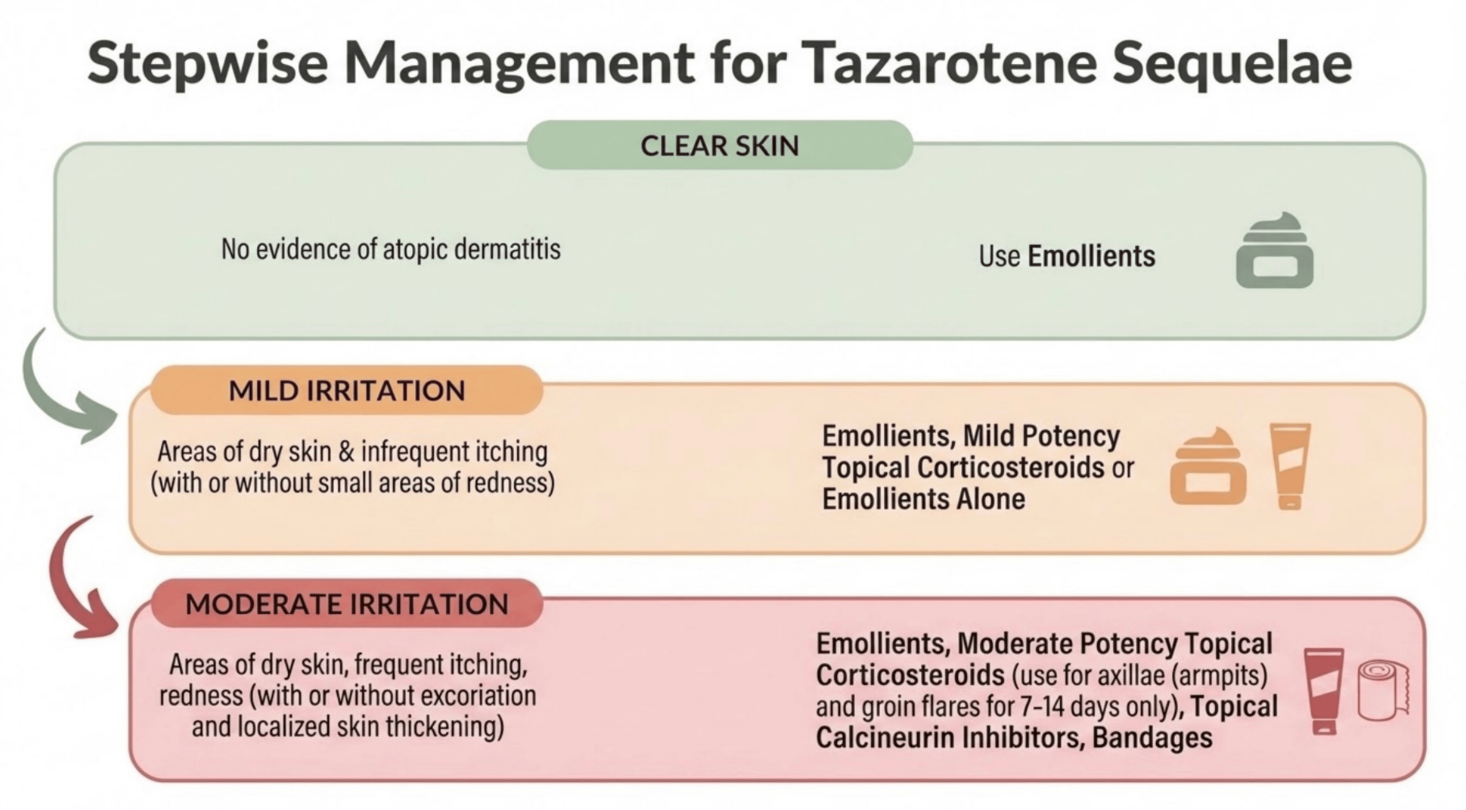
Stepwise management of tazarotene-induced sequelae Suggested management pathway for persistent tazarotene-associated facial erosions: discontinuation of tazarotene, emollients and photoprotection, short-course topical corticosteroids when appropriate, laboratory exclusion of inflammatory mimics when clinically indicated, and transition to topical pimecrolimus in refractory facial disease. This figure was created by the authors using Microsoft PowerPoint (Microsoft Corporation, Redmond, Washington, United States). No external copyrighted materials, artificial intelligence (AI) tools, or large language model (LLM) tools were used in the creation or modification of this figure.

This case illustrates the importance of excluding mimicking conditions without misinterpreting negative systemic testing. The patient's negative ANA and normal ESR and CRP levels suggested no underlying systemic autoimmune process; however, this did not diminish the severity of the localized cutaneous inflammation. Instead, these findings reinforced the appropriateness of a localized treatment strategy. A stepwise management plan is summarized in Figure 2, beginning with withdrawal of the offending retinoid and photoprotection, followed by limited corticosteroid use when appropriate, and transitioning to calcineurin inhibition in refractory facial disease.

**Figure 3:**
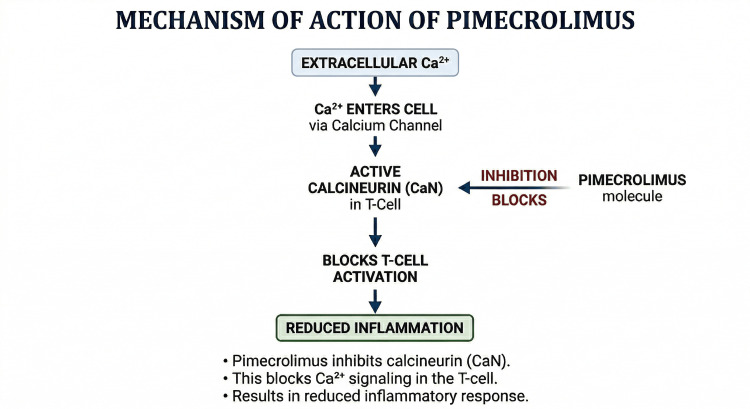
Mechanism of action of topical pimecrolimus in T-cell-mediated inflammation Extracellular calcium (Ca²⁺) enters the T cell through membrane calcium channels, contributing to the activation of calcineurin (CaN), a calcium-dependent phosphatase involved in T-cell signaling. Pimecrolimus, a topical calcineurin inhibitor, binds to intracellular immunophilin macrophilin-12 (FKBP-12), and the resulting complex inhibits calcineurin activity. This prevents downstream T-cell activation and reduces the transcription of pro-inflammatory cytokines, thereby decreasing cutaneous inflammation. This simplified schematic is intended for the conceptual illustration of the anti-inflammatory effect of pimecrolimus. This figure was created by the authors using Microsoft PowerPoint (Microsoft Corporation, Redmond, Washington, United States). No external copyrighted materials, artificial intelligence (AI) tools, or large language model (LLM) tools were used in the creation or modification of this figure.

A schematic summary of the pimecrolimus mechanism is shown in Figure 3. By inhibiting calcineurin, pimecrolimus prevents the transcription of pro-inflammatory cytokines downstream of T-cell activation, which likely explains its efficacy in persistent facial inflammation when barrier-directed therapy alone is insufficient [4,5].

Several limitations should be acknowledged. This is a single-patient case report and therefore cannot establish comparative efficacy or generalizable treatment recommendations. No biopsy was performed, so the precise histopathologic substrate of the erosions could not be definitively confirmed. In addition, while the chronology strongly supports a retinoid- and ultraviolet-associated inflammatory reaction, causality remains clinical rather than experimentally proven. Finally, spontaneous delayed recovery after retinoid withdrawal cannot be excluded with certainty, although the lack of improvement over six months prior to pimecrolimus initiation and the subsequent temporal response argue in favor of a therapeutic effect.

Overall, this case supports topical calcineurin inhibition as a reasonable steroid-sparing option in selected patients with persistent retinoid-associated inflammatory facial injury, particularly when standard supportive measures and topical corticosteroids have failed or when prolonged steroid exposure on facial skin is undesirable.

## Conclusions

Tazarotene-induced photosensitivity may, in rare cases, lead to persistent inflammatory facial erosions refractory to conservative measures and topical corticosteroids. This case demonstrates the successful resolution of treatment-resistant lesions with topical pimecrolimus and highlights the value of calcineurin inhibition as a steroid-sparing therapeutic option on facial skin.

Early consideration of topical calcineurin inhibitors may facilitate healing, reduce pruritus, restore barrier function, and limit corticosteroid-related adverse effects in chronic retinoid-associated facial inflammation. Additional reports are needed to better define which retinoid-induced eruptions are most likely to benefit from this approach.
